# Assessment of human health risks to tick-borne infections in urban green spaces (UGS) - a study protocol

**DOI:** 10.1186/s12879-025-12364-6

**Published:** 2025-12-24

**Authors:** Sibaram Sadangi, Marijo Parčina, Nico T. Mutters, Timo Falkenberg

**Affiliations:** 1 Institute for Hygiene and Public Health, University Hospital Bonn, Bonn, Germany; 2 Institute of Medical Microbiology, Immunology and Parasitology, University Hospital Bonn, Bonn, Germany

**Keywords:** *Borrelia*, *Rickettsia*, Tick-borne encephalitis virus, Tick-borne diseases, Vector-borne diseases, *Ixodes ricinus*

## Abstract

**Background:**

As the world becomes increasingly urbanised, with projections of about two-thirds of a burgeoning population living in urban areas by 2050, several governments are continuously making efforts to create, maintain, and expand urban green spaces and enhance their ecological connectivity. However, the advantages of these green infrastructures are weighed down by their risk of higher human exposure to wildlife, zoonotic diseases, and wildlife-associated ectoparasites, leading to spillage of vector-borne illnesses.

**Methods/design:**

The research study aims to examine the risk of acquiring tick-borne infections in various urban green spaces. The study aims to examine the impact of landscape features, habitat types, and host communities on the presence, abundance, and distribution patterns of ticks and tick-borne pathogens in cities’ green spaces. Simultaneously, it aims to assess the health risks due of tick-bites arising from human interaction within green spaces. Ticks will be collected from March to October in 2024 and 2025 through the flagging/dragging method at eleven green spaces in Germany’s Bonn and Cologne regions. Tick specimens will be examined for tick-borne pathogens. Simultaneously, an In-field survey will be conducted in these green spaces to assess green space users’ knowledge, attitude and practice behaviours concerning ticks and tick-borne pathogens.

**Discussion:**

Our research study will assess the suitability of urban green spaces with varying host communities, landscape composition and connectivity scales for sustaining tick populations and potentially transmitting tick-borne pathogens to humans. We aim to quantify the risk of acquiring tick-borne infection in urban green spaces and formulate recommendations for designing and managing green infrastructures to reduce the transmission of tick-borne pathogens to humans.

**Study registration:**

Clinical trial number: not applicable.

**Supplementary Information:**

The online version contains supplementary material available at 10.1186/s12879-025-12364-6.

## Background

Vector-borne diseases (VBDs) constitute 17% of the estimated global burden of all infectious diseases, leading to more than 700,000 deaths annually [1]. Approximately 6.3 billion people are at risk of vector-borne illnesses, leading to 300 million cases annually [2, 3]. Tick-borne diseases (TBDs) constitute a disproportionately high share of all VBDs reported in North America [4] and Europe [5, 6]. The arachnid vectors are obligate hematophagous ectoparasites that transmit several pathogens of medical and veterinary importance. The predominant tick species in Europe, *Ixodes* (*I.*) *ricinus*, can transmit a wide array of pathogens, many of which (more than 20) are implicated in human illnesses. Some widely known diseases in humans and animals include Lyme Borreliosis (LB), tick-borne rickettsioses, anaplasmosis, babesiosis, relapsing fever, and tick-borne encephalitis (TBE) [7, 8].

The incidence of TBDs is increasing due to multiple factors, such as the expanding geographical range of vectors, changes in land use, reforestation, habitat fragmentation, and climate change, among others [6, 7, 9]. Cases of TBDs have more than doubled in the U.S. in the last two decades [4, 10]. In recent decades, Europe has also witnessed an increasing trend in the prevalence of TBE and bacterial TBDs, including LB [7, 8, 11]. The diagnosis, management and treatment of TBDs are hindered by low awareness of emerging diseases and diagnostic inefficiency due to nonspecific clinical symptoms [12, 13]. Prevention relies on protective measures against tick bites and immediate removal of the tick post-bite, as vaccination is only available against TBE [12, 14].

As urbanisation transforms the world, it is projected that by 2050, nearly 68% of the global population (around 6.68 billion people) will reside in urban areas, compared to 46.6% in 2000 [15, 16]. Building healthy urban societies requires integrated management of human societies and the local ecosystem. A promising initiative adopted in this direction is integrating green spaces to advance healthy lifestyle choices among the urban population and halting biodiversity loss [8, 15]. However, Urban Green Spaces (UGS) raise a few health concerns, including pollen-induced allergies [17] and respiratory problems [18] or potential breeding grounds for vectors that may facilitate disease spillover from wildlife or zoonotic diseases, among others [19, 20]. In the past decade, studies on ticks have underscored the risk of acquiring tick-borne pathogens (TBPs) in cities [21]. The prevalence of ticks and tick-borne infections among questing ticks in UGS is similar to rural areas [22–24], thereby increasing the risk of acquiring infections in UGS due to the higher frequency of human visits to UGS [21, 25].

Several factors influence the presence, abundance, and distribution of ticks and TBPs in green spaces, including landscape connectivity and composition, abundance of host species, and local habitat types [6, 19, 22, 26, 27]. However, the impact of these factors on the distribution of vectors and pathogen transmission along an urbanisation gradient is poorly deciphered [19, 27]. This research study aims to evaluate the impact of three key factors: (1) landscape connectivity, (2) host community, and (3) landscape composition on the abundance and activity of ticks.

Landscape connectivity sheds light on the degree of linkage between two green spaces, i.e. the flow of host species from one green space to another and their interactions with both environments [28]. We hypothesise that connectivity between green spaces facilitates the movement of animals, which may introduce ticks to a green space or increase the number of hosts for the tick population in a green space, thereby playing a crucial role in introducing, establishing, and/or amplifying tick populations. This will influence TBP circulation in the enzootic cycles of connected green spaces [26, 29].

The host community (small and medium-sized mammals, dogs and cats, roe deer, wild boar, and birds) found in urban and peri-urban areas can be maintenance hosts for ticks and important reservoirs of TBPs [30, 31]. Several research studies have correlated a higher abundance of tick populations with higher deer presence [32] and abundance [33–35]. Changes in host preferences for animals commonly found in urban ecosystems, such as hedgehogs, foxes, hares, and companion animals, can alter the diversity and prevalence of TBPs [30]. The increasing presence of deer and wild boar in urban settings leads to viable tick populations, a shift in enzootic cycles of associated pathogens, and increased health risks for humans [30, 31].

Landscape composition and configuration (proportion and spatial arrangement of habitat types) play a significant role in moderating the environmental conditions, host movement, host community, and habitat usage in a green space required for tick survival and development, resulting in differences in tick abundance across habitat types [36, 37].

The risk of acquiring tick-borne infections is influenced by the “hazard” posed by the entomological risk and “exposure” to the hazard. The exposure is dependent upon the green space users’ usage of the green space, knowledge, risk perception, and preventative behaviours regarding tick bites [8, 38, 39]. Integrating socio-demographic studies with tick surveillance in cities is important since UGS is frequented by many visitors who primarily perceive a low risk of tick bites in urban areas. Therefore, this research study adopts a holistic approach to measuring health risks of tick-borne infections in UGS by (1) identification, understanding, and prediction of the abundance and geographic distribution of ticks and TBPs and (2) understanding socio-demographic factors influencing exposure to tick bites in green spaces in cities.

## Methods/design

### Aims and objectives

UGS may pose potential health risks by exposing humans to TBPs circulating in the enzootic cycles of green spaces. Our research study aims to:

### Objectives

1. Assess the impact of landscape composition, connectivity and host communities on the suitability of UGS for tick and TBP inhabitation.
2. Investigate the dynamics of tick-borne pathogen infection rates across different UGS.
3. Evaluate the risk of potential transmission of tick-borne infections to humans across different UGS and determine the factors influencing these human health risks.

### Research questions

1. How does tick abundance vary among green spaces with differing landscape compositions, levels of connectivity, and host communities?
2. What is the diversity and prevalence of tick-borne pathogens in UGS?
3. What is the risk of humans acquiring tick-borne infections in urban areas?

### Study setting

**Fig. 1:**
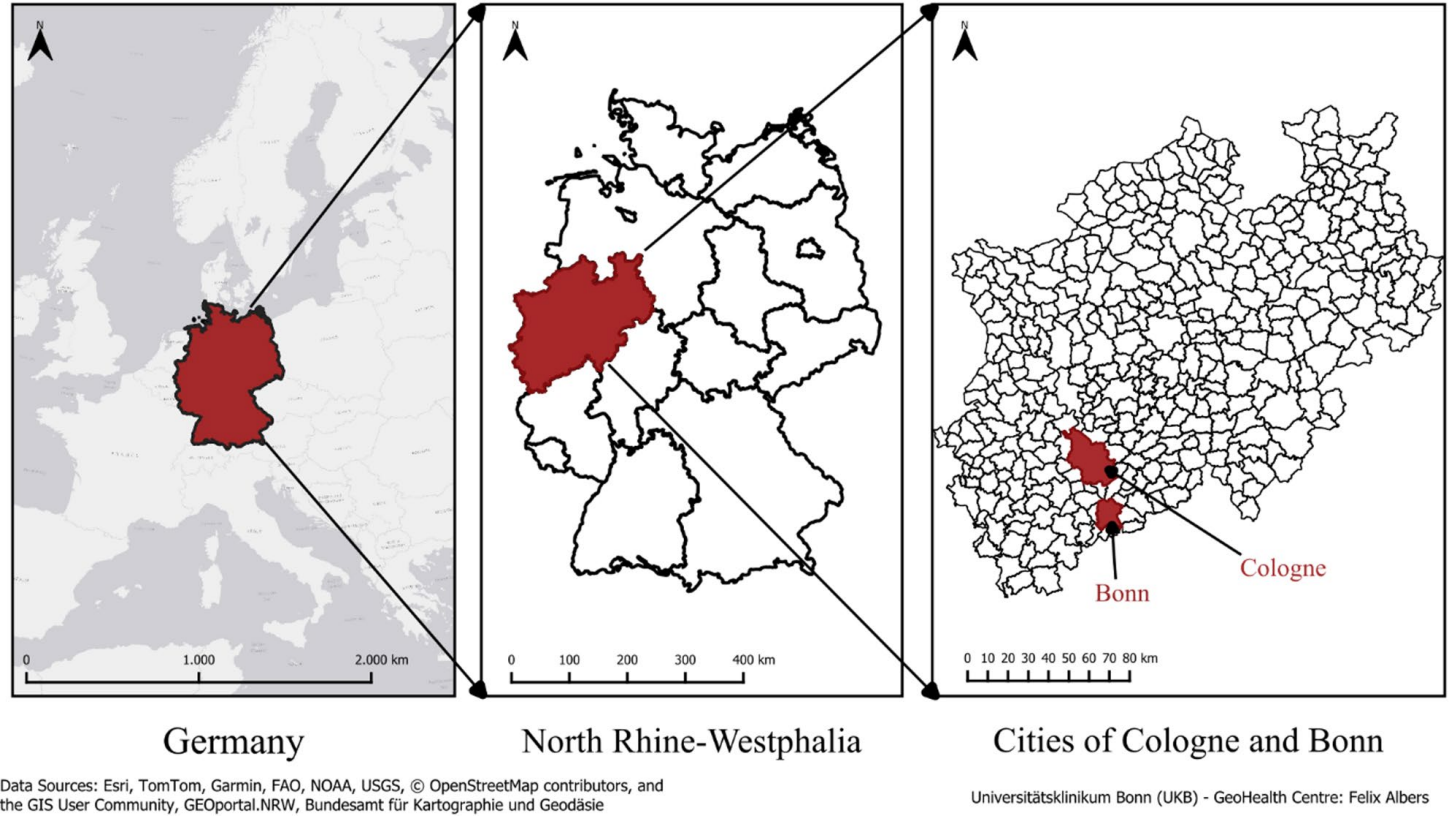
Bonn and Cologne Cities of Germany: study site for the research study

The research study is set in Bonn and Cologne, North-Rhine Westphalia (NRW), Germany, as shown in Fig. 1. Bonn and Cologne have 335,789 and 1,092,520 inhabitants (as of 31 December 2023), respectively [40, 41]. In seven out of 16 federal states of Germany, including North-Rhine Westphalia State, reporting for LB cases is not mandatory [42].

### Research design

**Fig. 2:**
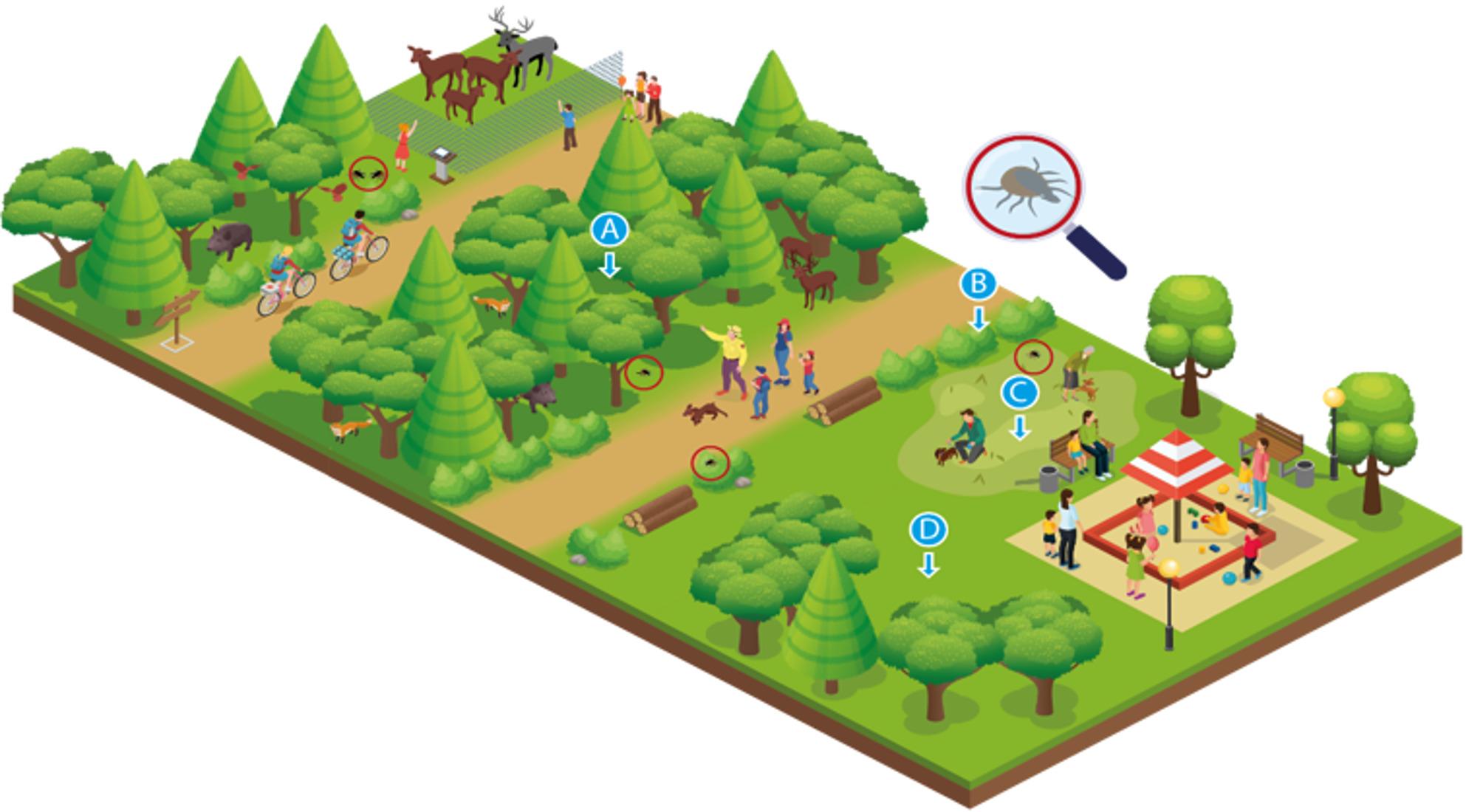
Illustration of UGS serving as shared environments for humans, wildlife, vectors, and pathogens. Ticks will be collected in public green spaces at 3–5 sampling spots, such as (**A**) Woodland, (**B**) Shrubs, (**C**) Unmowed land and (**D**) Mowed land, representing various sites humans interact with while using green spaces. Additionally, ticks will be collected at dog-walking areas if present in the green space

The study is set in public green spaces of the Bonn-Cologne region frequently visited by residents in daily life and for weekend activities. A wide array of activities, ranging from walking, jogging, or meeting friends and family to visiting animal reserves or hiking, are practised in these green spaces. UGS, as depicted in Fig. 2, serve as shared environments where humans, host animals, and vectors coexist. While these areas provide ecological and recreational benefits, they also facilitate interactions between wildlife, vectors and pathogens, potentially increasing the risk of zoonotic diseases.

**Fig. 3:**
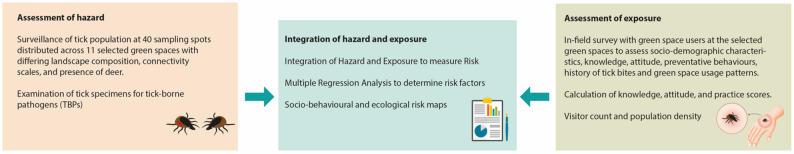
Framework for assessment of human health risks to tick-borne infections in urban green spaces (UGS)

The research study observes the complex issue of the risk of acquiring tick-borne infections in UGS through a socio-ecological lens. The study aims to understand the risk of acquiring tick-borne infections in UGS through (1) assessment of hazard, (2) assessment of exposure, and (3) integration of hazard and exposure variables to calculate risk, as conceptualised in Fig. 3. Figure 3 illustrates the research design of the longitudinal risk-assessment study. As depicted in the left box, the hazard will be assessed by determining the entomological hazard posed by ticks in UGS and characterizing the environmental factors (such as landscape composition, connectivity, host biodiversity, and microclimate) influencing the hazard, for which questing ticks will be collected at several sites, with varying landscape features, habitat types and host biodiversity and then examined in the laboratory for infection rates with TBPs. Assessment of the exposure will be done as illustrated in the right box, by counts of visitors, population density around the green space, and a Knowledge-Attitude-Practice (KAP) survey with green space users in the UGS. As depicted in the middle box, hazards and exposure will be integrated to model the risk posed by UGS in potentially transmitting TBPs to green space users.

### Selection of green spaces and sampling spots

The presence, abundance, and distribution of *I. ricinus* ticks vary temporally and spatially, depending upon a multitude of factors such as habitat type, availability of hosts, and abiotic factors such as landscape characteristics and microclimate [22, 43]. We hypothesise that landscape composition, connectivity, and host biodiversity of UGS play an important role in the abundance of ticks and the prevalence of associated pathogens. The green spaces and sampling spots in Bonn and Cologne have been selected to represent UGS with differing landscape connectivity, composition, host community, and habitat types to study their impact on tick abundance, prevalence of TBPs, and human health risks.

**Fig. 4:**
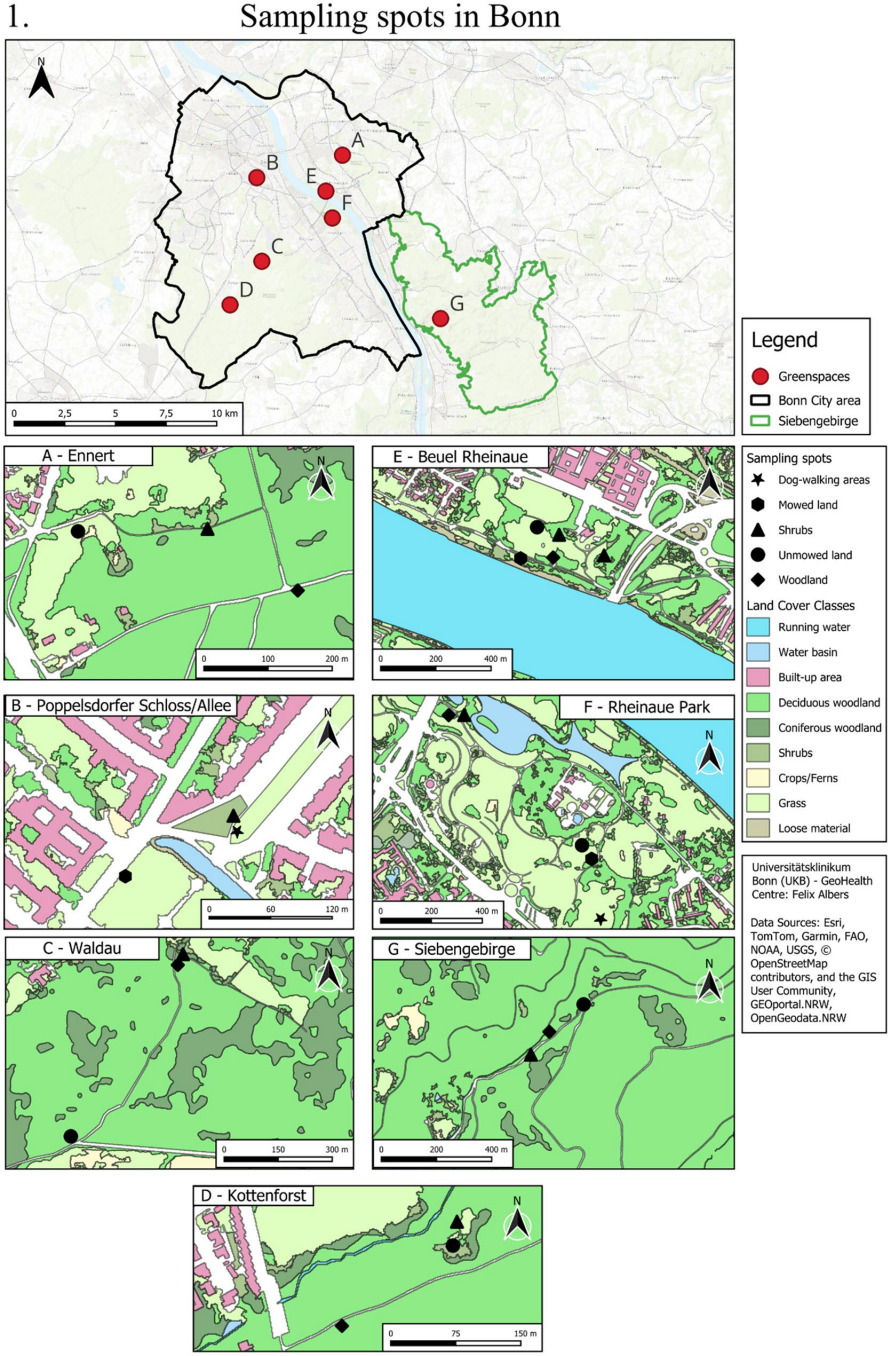

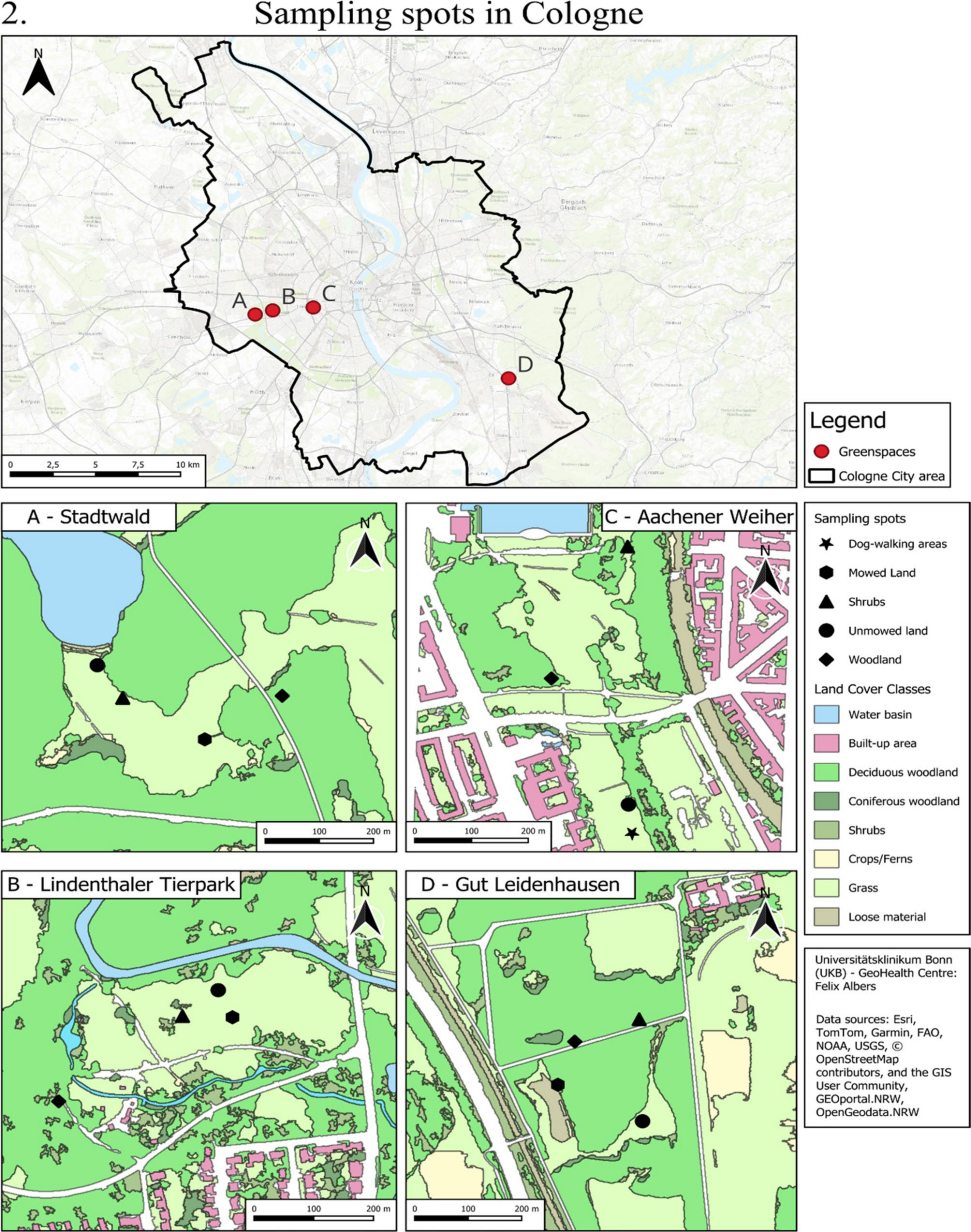
Ticks will be collected at sampling spots representing woodland, shrubs, mowed land, unmowed land, and dog-walking areas structures in (1) seven green spaces in Bonn and (2) four green spaces in Cologne

In total, eleven green spaces (seven in Bonn and four in Cologne, as shown in Fig. 4), broadly categorised into forests (*N* = 3: 2 urban forests; 1 peri-urban forest), green spaces connected to/ in the vicinity of forests (*N* = 4), and green spaces not connected to/separated from the other green spaces by urban infrastructure (*N* = 4), have been selected as study sites for tick surveillance and exposure assessment, as detailed in Table 1. The selected green spaces have varying presence of deer, broadly classified into four categories, as described in Table 1: (i) absence of deer (*N* = 4), (ii) presence of captive deer (*N* = 2), (iii) presence of free-roaming deer (*N* = 3) and (iv) presence of both free-roaming and captive deer (*N* = 2). Information on deer presence was obtained through direct personal observations made during tick collection activities and was corroborated through informal interviews with green space management officials. In each green space, ticks will be collected from a total of three to five sampling spots. Each spot represents a distinct and prevalent habitat type within the green space (e.g. woodland, shrubs, unmowed land, mowed land, or dog-walking areas). Each habitat type is represented by one sampling spot in a green space, and the total number of spots per green space reflects the diversity of habitat types present, typically ranging from three to five. These sampling spots are selected in relative proximity within the green space to minimise differences in the environmental conditions, apart from the vegetation structure they represent. While the woodland habitat characteristically contains dead leaf litter and twigs-cover lying under a continuous stretch of trees (of at least 20 m^2^), shrub vegetation up to a height of 1 m, present at the edge of woodland or alone constitutes the shrub sampling spot. Ticks will also be collected at unmowed land habitat type (areas with grass up to a height of 1 m, left unmowed for a time of the year) and mowed land covers (areas constantly mowed by the city administration as a part of management practices). In addition, ticks will be collected at dog-walking areas within UGS. The research study encompasses 40 sampling spots (10 Woodland, 11 shrubs, 10 unmowed land, 6 mowed land, and 3 dog-walking areas) to monitor ticks and associated pathogens, as shown in Fig. 4.

**Table 1:** Selection of green spaces with varying levels of landscape connectivity, composition and deer presence: (i) absence of deer (-), (ii) presence of deer in captivity (+), (iii) presence of free-roaming deer (++) and (iv) presence of both free-roaming and deer in captivity (+++)

Types of Green Spaces	Group 1 (Bonn)	Group 2 (Bonn)	Group 3 (Cologne)	Additional green spaces
Forest (*N* = 3)	Siebengebirge (++)	Kottenforst (++)	Stadtwald (-)	
Green Space connected to/ in the vicinity of Forest (*N* = 4)	Ennert (++)	Waldau (+++)	Lindenthaler tier park (+)	Gut Leidenhausen (Cologne) (+++)
Green Space separated from other green spaces (*N* = 4)	Beuel Rheinaue Park (-)	Poppelsdorf Schloss/Allee (-)	Aachener Weiher (-)	Bonn Rheinaue Park (+)

## Methods for assessment of hazard

### Collection of tick specimens

**Fig. 5:**
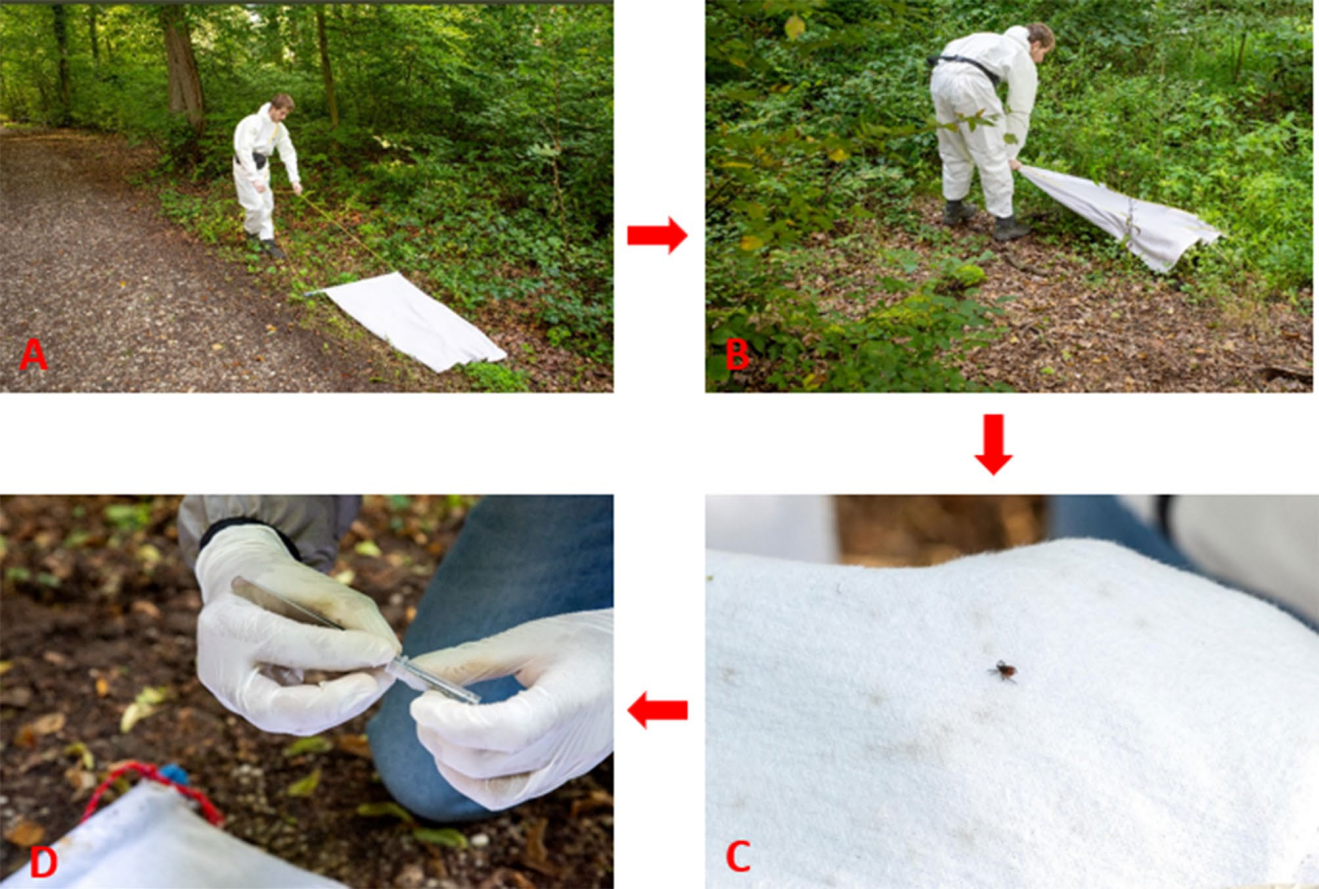
Collection strategy of tick sampling: **A**) dragging the flag at woodland vegetation, **B**) flagging the shrub vegetation, **C**) gripping of tick specimens to the fabric and **D**) storage of tick specimens in lysis buffer. Photo courtesy: Katharina Wislsperger of University Hospital Bonn

The flagging/dragging method of tick collection will be used in this research study for the surveillance of tick abundance, pathogen prevalence, and assessing host-seeking phenology [44, 45]. Questing ticks will be collected by dragging and/or flagging a 1 m² cloth over a 100 m² area at each sampling spot [46, 47]. The 1m^2^ flag is supported by a metal dowel rod sewn into one end to which the rope is tied. The rope will be dragged or flagged (horizontally or vertically) along the vegetation structures (as depicted in Fig. 5), allowing for maximum contact of the flag with the sampling spot [48]. The tick specimens grip the cotton texture of the flag. The flags will be inspected on both sides for tick specimens after every 3 m of flagging. In case of any doubts in recognising a specimen, a magnifying glass will be used.

From March to October in 2024 and 2025, tick and pathogen surveillance will be conducted monthly on clear days between 9:00 a.m. and 2:00 p.m. in 11 urban green spaces. Within each green space, 3 to 5 sampling spots (300–500 m²) will be surveyed monthly [45, 46], resulting in a total of 40 spots across 11 green spaces, covering approximately 4,000 m². The spots are distributed across five habitat types: 10 woodland spots (1,000 m²), 11 shrubland spots (1,100 m²), 10 unmowed land spots (1,000 m²), 6 mowed land spots (600 m²), and 3 dog-walking areas (300 m²). This design enables standardised and high-resolution temporal monitoring of tick presence and associated pathogens across diverse urban habitats. The time duration of March to October was selected to take into account the higher activity of *I. ricinus* ticks in these months compared to winter months in Germany [49]. The study aims to account for the bimodal activity of *I. ricinus* ticks, as reported in several studies [50, 51], with an initial peak in the spring followed by a lower peak in the autumn. The characteristics of tick collection in this research study are outlined in Table 2; Fig. 5. Temperature and relative humidity will be measured with a Trotec BC06 Thermohygrometer at ground level before and after tick collection at each sampling spot to consider their critical role in the abundance of ticks. The average of the two recordings will be used to indicate the microclimatic condition of each collection.

**Table 2:** Characteristics of collection of tick specimens at the selected green spaces in Bonn and Cologne regions in Germany (Duration: March-October 2024 and 2025)

Study setting	Bonn and Cologne regions of Western Germany
Number of selected green spaces in Bonn and Cologne regions	11
Number of selected sampling spots	40 (3–5 per green space)
Area of a sampling spot surveyed every month	100 m^2^
Collection strategy	Dragging/Flagging a 1 m^2^ white cotton cloth
Collection time	9 am to 2.0 pm
Collection weather	Clear day, no rain
Other measured parameters during the collection time (apart from tick abundance)	• Microclimate (relative humidity, temperature) at each sampling spot • Number of humans • Number of dogs • Collection of flies for assessment of mammalian biodiversity (July-September 2024 and April-July 2025)
Storage of ticks	Guanidine thiocyanate-based media at 4 °C

### Storage of tick specimens

While larvae specimens will be pooled in batches of 3–12 per collection, nymph and adult samples will be collected individually. All the distinct tick specimens (larvae, nymph, and adults) collected will be stored in 450 µL of guanidine thiocyanate-based media, labelled with the date, sampling spot, and site of collection, and stored at 4 °C until further examination. The guanidine thiocyanate-based media is a strong protein denaturant and prevents nucleic acid degradation by nucleases, ensuring its stability for molecular diagnostics [52, 53].

### Analysis of tick specimens for determining the prevalence of tick-borne pathogens

**Fig. 6:**
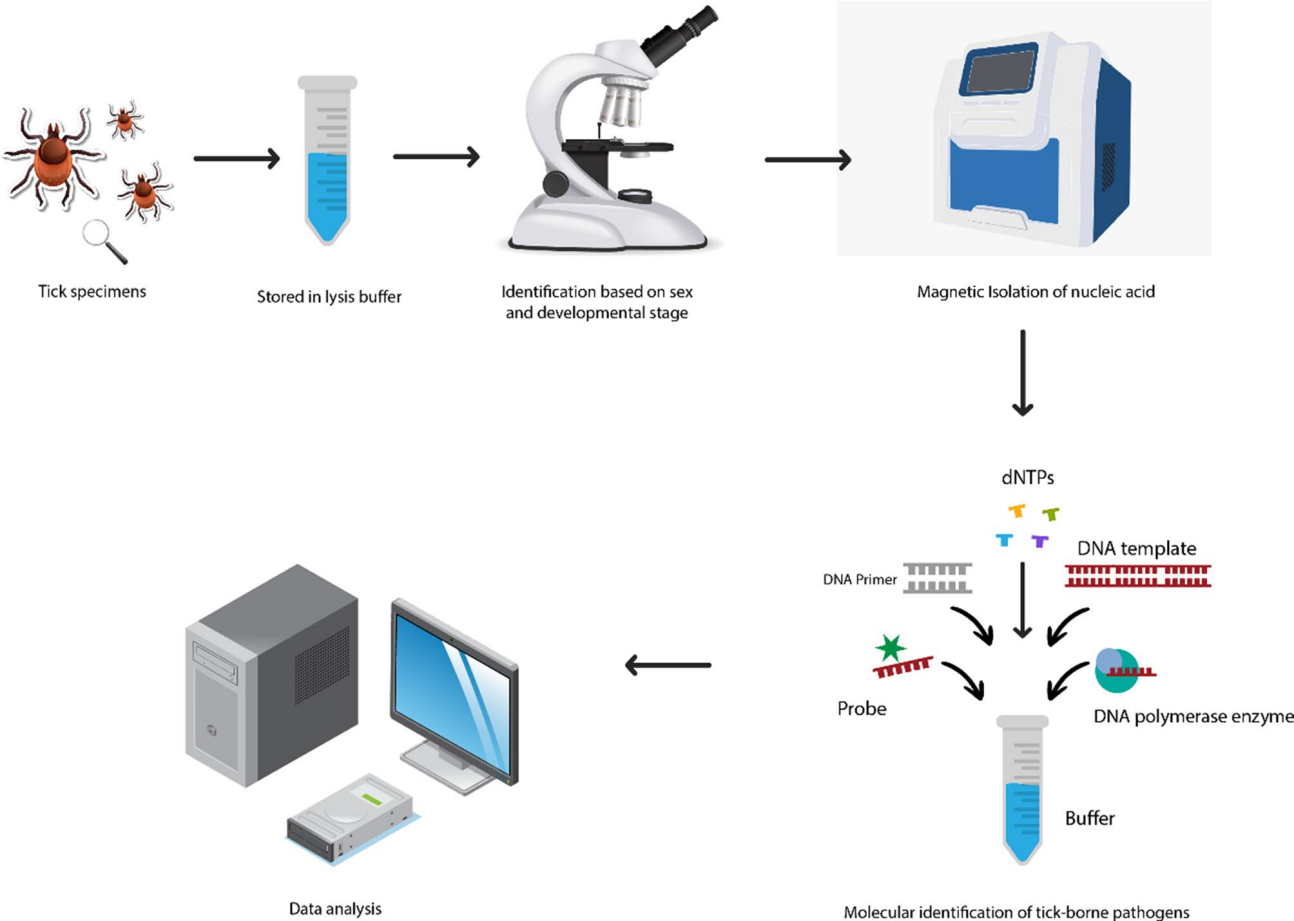
Flowchart of the steps to analyse tick specimens collected at the eleven green spaces in the laboratory

Tick specimens collected for entomological surveillance will undergo morphological identification, and the prevalence of tick-borne pathogens will be analysed in the laboratory according to the workflow outlined in Fig. 6. The individual steps of the process are described in detail below.

### Identification of tick specimens using taxonomic keys

The tick specimens will be differentiated based on their developmental stage (larvae, nymph, and adult) and gender (adult male and adult female). The tick specimens will be simultaneously identified morphologically up to species level using taxonomic keys under microscopic observation [54].

### Pre-analytic processing of tick specimens prior to nucleic acid extraction

Following the identification of ticks using taxonomic keys, individual tick specimens (including larvae pools) will be crushed with sterile plungers to fragment the chitin exoskeleton [55] and release the intracellular components into the lysis buffer (guanidine thiocyanate-based media). Subsequently, 30 µL of recombinant-proteinase K will be added to individual tick specimens, followed by overnight incubation at 56 °C, with continuous shaking [56].

### Isolation of nucleic acid from tick specimens

Tick specimens in batches of 96 samples will be processed to isolate nucleic acid using Chemagic 360 Instrument following the manufacturer’s instructions. Total nucleic acid isolation through Chemagic 360 Instrument utilises magnetic beads to separate and purify genetic material from the tick specimens. Specifically, we will employ the chemagic *Body Fluid 200 360 H96 prefilling VD220531.che* protocol provided by Revvity to extract genetic material from 200 µL of tick suspension (containing 150 µL of tick specimen and 50 µL of Dulbecco’s Phosphate Buffered Saline) [57]. The isolated nucleic acid will be stored at -20 °C for short-term preservation or at -80 °C for long-term storage (> 2 weeks) to maintain RNA stability, which is necessary for TBE RNA detection in tick specimens.

### Molecular identification of *I. ricinus* ticks

*Ixodes ricinus* ticks are the most abundantly reported tick vector in our region [51, 58, 59]. However, to avoid misidentification with closely related species like *I. inopinatus*, *I. hexagonus*, or *I. persulcatus*, molecular identification of *I. ricinus* tick specimens will be done using real-time PCR. If the specimen is not identified as an *I. ricinus* tick, the amplified PCR product will be Sanger-sequenced to determine the species type.

PCR reactions will be set up in an end volume of 20 µL, which includes 10 µL of Promega GoTaq^®^ Probe qPCR Master Mix, 10 µM forward primer, and reverse primer each (IXO-I2-F4 and IXO-I2-R4) to amplify the *Ixodes* spp. *ITS2* gene, 2.5 µM of Iri-12-P4 probe (specific for *I. ricinus*) (Table 4), 5µL of nucleic acid and remaining nuclease-free water, using the thermal set-up as detailed in Table 3 in the Bio-Rad CFX96 thermal cycler.

**Table 3:** Thermal cycling profile of the PCR amplification process for identification of *I. ricinus* ticks

Step	Temperature (°C)	Time	Cycles
GoTaq^®^ DNA Polymerase activation	95	2 min	1
Denaturation	95	5 s	40
Annealing and elongation	60	30 s	

**Table 4:** Genetic sequences of primers and probes used to detect tick and tick-borne pathogens, manufactured by microsynth GmbH, Germany

Target	Primer/Probe Name	Primer/Probe Target (5’ → 3’)	Reference
Ticks 16 S rRNA	Tick 16 S-F	CTGCTCAATGATTTTTTAAATTGCTGTGGT	[60]
	Tick 16 S-R	CCGGTCTGAACTCAGATCAAGTAGGA	
	Tick16S-P	[Cy5]-AAATAGTTTGCGACCTCGATGTTGGATTAGGAT-[BHQ-2]	
*Ixodes ricinus* *ITS2*	IXO-12-F4	TCTCGTGGCGTTGATTTGC	[61]
	IXO-12-R4	CTGACGGAAGGCTACGACG	
	Iri-12-P4	[VIC]-TGCTCGAAGGAGAGAACGA-[BHQ1]	
*B. burgdorferi* s.l. flagellin gene (*flaB*; p41)	FlaF1	AGCAAATTTAGGTGCTTTCCAA	[62, 63]
	FlaR1	GCAATCATTGCCATTGCAGA	
	FlaP	[FAM]- TGCTACAACCTCATCTGTCATTGTAGCATCTTTTATTTG -[BHQ1]	
*B. miyamotoi* flagellin gene (*flaB*; p41)	Bmp41F	TTGCTTGTGCAATCATAGCC	
	Bmp41R	GCAAATCTTGGTGCTTTTCAA	
	Bmp41P	[HEX]-AGATGCCACAATTTCATCTGTCATTA -[BHQ1]	
*Rickettsia* spp. 23 S rRNA	PanR8_F	AGCTTGCTTTTGGATCATTTGG	[64]
	PanR8_R	TTCCTTGCCTTTTCATACATCTAGT	
	PanR8_P	[ROX]- CCTGCTTCTATTTGTCTTGCAGTAACACGCCA –[BHQ-2]	
*Babesia* spp. 18 S rRNA	Bab18S-F	CAGCTTGACGGTAGGGTATTGG	[65–68]
	Bab18S-R	TCGAACCCTAATTCCCCGTTA	
	Bab18S-P	[ROX]-CGAGGCAGCAACGG-[BHQ2]	
*Anaplasma* spp. *Msp2*	ApMSP2-F	ATGGAAGGTAGTGTTGGTTATGGTATT	
	ApMSP2-R	TTGGTCTTGAAGCGCTCGTA	
	ApMSP2-P	[FAM]-TGGTGCCAGGGTTGAGCTTGAGATTG-[BHQ-1]	
TBEV non-struct. prot. 5	TBE1-F	GGGCGGTTCTTGTTCTCC	
	TBE1-R	ACACATCACCTCCTTGTCAGACT	
	TBE1-P	[Cy5]-TGAGCCACCATCACCCAGACACA-[BHQ-2 ]	
*Borrelia* IGS	B5Sborseq-F	GAGTTCGCGGGAGAGTAGGTTATTGCC	[69–71]
	23Sborseq-R	TCAGGGTACTTAGATGGTTCACTTCC	
*Rickettsia* spp. *gltA*	CS877-F	GGGGACCTGCTCACGGCGG	
	CS1258-R	ATTGCAAAAAGTACAGTGAACA	

### Examination of tick specimens for tick-borne pathogens

In this research study, the tick specimens will be examined for *Rickettsia* spp., *Borrelia miyamotoi*,* Borrelia burgdorferi* s.l., *Anoplasma phagocytophilum*, and *Babesia* spp. In addition to the circulating pathogens in our region, we are examining the tick specimens for tick-borne encephalitis as part of routine surveillance measures, keeping in mind the increase in TBE cases observed in Germany from 2001 to 2018, wherein the risk areas have increased from 129 districts in 2007 to 161 in 2019, an expansion observed in southern, north-eastern and patchy north-western parts of Germany [11]. Aliquots of nucleic acid samples from 10 tick specimens will be pooled to detect *Anaplasma phagocytophilum* DNA, *Babesia spp.* DNA, and tick-borne encephalitis virus RNA since the prevalence of these pathogens is relatively low in our region (unpublished data). The genetic sequences of primers and probes to be used in this research study is detailed in Table 4. While specimens previously confirmed as positive specimens will be employed as positive controls in the qPCR assays, nuclease-free water will be used as the negative control in every PCR run. All samples will be analysed in two replicate reactions. A sample will be considered positive for a pathogen only when it is determined to be positive in both replicate reactions. All the PCR reactions will be set up in the Bio-Rad CFX96 thermal cycler and analysed with Bio-Rad CFX96 Manager IVD Edition 1.6.

### Detection of *Borrelia* spp. and *Rickettsia* spp. DNA in tick specimens

The detection of *Borrelia* spp. *(B. burgdorferi* s.l., and *B. miyamotoi)*, and *Rickettsia* spp. DNA will be done using an in-house multiplex q-PCR assay. PCR reaction, prepared in volumes of 20 µL by combining 10 µL of Promega GoTaq™ Probe qPCR Master Mix, 10 µM of forward and reverse primers (FlaF1, FlaR1, Bmp41F, Bmp41R, PanR8_F, and PanR8_R) targeting the flagellin (*flaB*; p41) gene in *Borrelia* spp. and 23 S rRNA in *Rickettsia* spp., 1 µM of tick primers (Tick 16 S-F, Tick 16 S-R), 2.5 µM of pathogen-specific probe (FlaP, Bmp41P and PanR8_P), 0.25 µM of tick probe (Tick16S-P) (Table 4), 5 µL of nucleic acid and remaining nuclease-free water, will be amplified in a Bio-Rad CFX96 thermal cycler using the thermal set-up specified in Table 5. The amplification of the tick 16 S rRNA gene will serve as an internal control in each PCR reaction. The presence of internal control, *B. burgdorferi* s.l., *B. miyamotoi*, and *Rickettsia* spp. DNA will be measured through Cy5, FAM, HEX, and ROX channels.

**Table 5:** The thermal profile to detect the presence of *B. burgdorferi* s.l., *B. miyamotoi*, and *Rickettsia* spp. With multiplex in-house PCR

Step	Temperature (°C)	Duration	Cycles
GoTaq^®^ DNA Polymerase activation	95	2 min	1
Denaturation	95	5 s	45
Annealing and elongation	60	30 s	

### Identification of *Borrelia burgdorferi s.l* and *Rickettsia* genospecies in tick specimens

The study seeks to investigate the genospecies of *Borrelia burgdorferi* s.l. and *Rickettsia* spp. circulating in the Bonn-Cologne region. In the samples found positive for *B. burgdorferi* s.l. and *Rickettsia* DNA by qPCR, genospecies differentiation will be done by sequencing the 5–23 S rDNA (rrfA-rrlB) intergenic spacer region (IGS) and *Rickettsia gltA* gene respectively. The PCR reaction will be prepared in a total volume of 20 µL containing 10 µL of Promega GoTaq™ qPCR Master Mix and 250 nM of primers each (B5Sborseq-F and 23Sborseq-R, or CS877-F and CS1258-R) (Table 4), 5 µL of nucleic acid and remaining nuclease-free water. The thermal cycling program will be run as specified in Tables 6 and 7.

**Table 6:** Thermal cycle for PCRs used to amplify the 5–23 S rDNA Intergenic spacer region for *B. burgdorferi* s.l. In positive specimens

Step	Temperature (°C)	Duration	Cycles
Activation	95 °C	2 min	1
Denaturation	95 °C	20 s	10
Annealing	72 °C → 60 °C (decreasing 1 °C each cycle)	30 s	
Elongation	72 °C	30 s	
Denaturation	95 °C	20 s	30
Annealing	60 °C	30 s	
Elongation	72 °C	30 s	
Final elongation	72 °C	7 min	1

**Table 7:** Thermal cycle for PCRs used to amplify *Rickettsia* spp. *GltA* gene in positive specimens

Step	Temperature (°C)	Duration	Cycles
Activation	95	2 min	1
Denaturation	95	10 s	45
Annealing	64	30 s	
Elongation	72	1 min	

The amplification will be monitored by the signal in the Syber-green (FAM) channel, and the specificity of the reaction will be monitored with High-Resolution Melting curve analysis. The purification of the PCR product will be done by GenUP™ PCR Cleanup Kit (biotechrabit, Berlin). The purified PCR product will be sequenced by Sanger sequencing, and the result will be run through BLAST to determine the species of *Borrelia* and *Rickettsia* genospecies circulating in the region.

### Identification of *Anaplasma phagocytophilum* and *Babesia* spp. DNA in tick specimens

A duplex qPCR will be used to determine the detection of *Anaplasma phagocytophilum* and *Babesia* spp. DNA in tick lysates by targeting *Msp2* and 18 S rDNA in *Anaplasma phagocytophilum* and *Babesia* spp., respectively. PCR reactions, prepared in volumes of 20 µL by combining 10 µL of GoTaq^®^ Probe qPCR Master Mix, 10 µM of forward and reverse primers (ApMSP2-F, ApMSP2-R, Bab18S-F and Bab18S-R) and 2.5 µM of pathogen-specific probe (Bab18S-P and ApMSP2-P) (Table 4), 5 µL of nucleic acid and remaining nuclease-free water, will be amplified in a Bio-Rad CFX96 thermal cycler using the thermal set-up specified in Table 8. The fluorescence for the presence of *Anaplasma phagocytophilum* and *Babesia* spp. will be measured in FAM and ROX channels.

**Table 8:** Thermal cycling profile for the duplex qPCR assay for detecting *Anaplasma phagocytophilum* and *Babesia* spp. DNA in tick lysates

Step	Temperature (°C)	Duration	Cycles
GoTaq^®^ DNA Polymerase activation	95	2 min	1
Denaturation	95	5 s	45
Annealing and Elongation	60	30 s	

### Identification of tick-borne encephalitis virus RNA in tick specimen

The qPCR master mix for TBEV detection in pooled nucleic acid samples will be prepared in a 20 µL reaction volume. PCR reaction, containing 10 µL of the GoTaq^®^ Probe qPCR Master Mix with dUTP, 0.4 µL of goScript™ RT mix for 1-step RT-qPCR, 10 µM primers (TBE1-F and TBE1-R), and 2.5 µM TBEV-specific probe (TBE1-P) (Table 4), 5 µL of nucleic acid and remaining nuclease-free water, will be amplified in a Bio-Rad CFX96 thermal cycler using the thermal set-up specified in Table 9. Fluorescence will be detected in the Cy5 channel.

**Table 9:** Thermal cycling profile for nucleic acid samples from tick specimens, used for detection of TBEV in tick specimens

Steps	Temperature (°C)	Duration	Cycles
Reverse Transcription	45	15 min	1
Reverse transcriptase inactivation and GoTaq^®^ DNA Polymerase activation	95	2 min	1
Denaturation	95	15 s	40
Annealing and elongation	60	60 s	

### Assessment of landscape composition and connectivity measures

Landscape composition and connectivity can be analysed using publicly available national [19, 27, 37] or international land cover datasets, including Corine Land Cover (CLC) [72–75]. These resources provide standardised, large-scale land use information suitable for ecological research and spatial planning. In our research study, the assessment of landscape composition and connectivity will be conducted using regional land cover data (Landbedeckung NRW Gesamt) available through OpenGeodata.NRW portal with a positional accuracy of 1–2 m. Among the 15 categories of land cover types, 12 landcover types relevant to our study sites classified into 5 major land cover types will be considered for final analysis: (1) **Woodland** consisting of deciduous and coniferous trees, (2) **Shrubs**: bushes and shrubs, (3) **Grass**: herbaceous vegetation – grass, crops, perennials, and Ferns (4) **Water Sources**: Inland Water and (5) **Impervious surfaces** consisting of loose material, solid rock and urban infrastructure such as high-rise buildings, and civil engineering structures. The proportion of these land cover types within three buffer zones (100 m, 500 m, and 1000 m) around the centroid of all the geo-coded sampling plots at each green space will be retrieved using QGIS. The impact of land cover types on tick presence and abundance will be studied at local (100 m), intermediate (500 m), and landscape levels (1000 m).

The assessment of landscape (or functional) connectivity will be done through the Least Cost Path Analysis (LCPA) on the land cover dataset. The method relies on modelling host animals’ ease or resistance as they move from one green space to another. Resistance scores will be assigned to land cover categories such as vegetation types and human infrastructures, which can either facilitate or obstruct the movement of organisms, mainly deer, across green spaces [19, 29]. The cumulative effort for animals (i.e. deer) to move from one green space to another will be measured in terms of Cost Distance using QGIS. In this way, each sampling area will be assigned a connectivity score, which will be used to assess the impact of connectivity on tick abundance. The higher the cost distance, the lower the connectivity of green spaces and vice-versa.

### Assessment of mammalian host community in green spaces

**Fig. 7:**
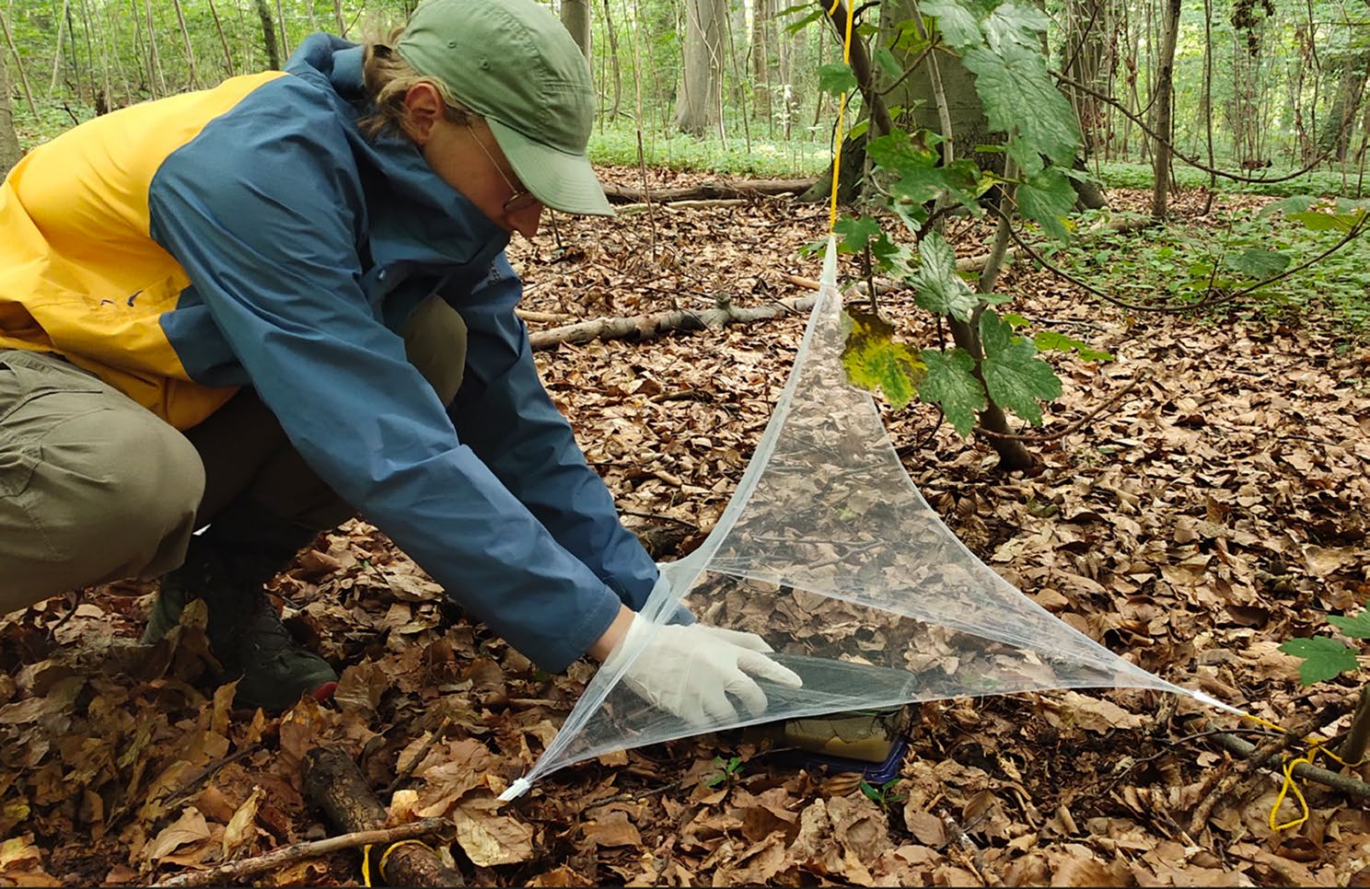
Fly trap set up to assess mammalian biodiversity in a green space. (Photo Courtesy of Sibaram Sadangi, University Hospital Bonn)

As we hypothesise that the host community of a green space influences its suitability to sustain tick populations and dispersal to other connected green spaces, it is necessary to determine the host composition of each green space. We will sample carrion flies and analyse the DNA they acquired while feeding on animal faeces or carcasses. Flies will be collected monthly from July to September in 2024 and April to July in 2025 using the fly trap method. Two artificial fly baits, covered by a net mesh to prevent direct contact with the flies, will be deployed in each green space, accompanied by a surrounding net trap, as shown in Fig. 7. The design allows flies to enter from the bottom while preventing their escape, ensuring effective capture. The artificial baits will be sourced from Feldner GmbH. The flies will be preserved in a falcon tube containing cotton soaked in 90% ethanol and stored at -20 °C. In the laboratory, nucleic acid will be extracted from the fly specimens, followed by PCR assays targeting mammalian genes [76–78]. The resulting PCR products will then undergo sequencing, enabling the taxonomic identification of the mammalian species based on the obtained genetic data [76–78].

### Statistical analysis

The statistical data analysis will be conducted using R. Analysis of tick density, pathogen prevalence, co-infection and environmental factors influencing tick abundance and TBP prevalence will be done using descriptive and inferential statistical methods. The significance threshold will be set at *p* < 0.05. The density of ticks (DO-Larvae, -Nymph, and -Adult) per 100 m^2^, as well as the prevalence of pathogens, will be determined across green spaces, habitat type, and month. Co-infection rates among pathogens will be computed for TBPs, and the co-occurrence of certain pathogens more frequently than others will be examined using Chi-Square or Fisher’s Exact Test. The monthly density of ticks will be compared between 2024 and 2025 using paired t-tests or Wilcoxon signed-rank tests, depending upon the normality of the data (to be assessed using the Shapiro-Wilk Test). Environmental factors such as cost distance, host composition (e.g. deer presence), land cover structure, habitat type, year, month, relative humidity, and temperature will be examined for their impact on tick abundance using Generalised linear mixed models (GLMMs). It is also planned to model entomological-risk zones within urban areas, which will be identified using the spatial risk modelling tool Maximum Entropy Modeling (MaxEnt). This habitat suitability prediction model will be incorporated with environmental variables, tick presence and abundance data, and infection prevalence to estimate high-risk areas for tick-borne infections.

## Methods for assessment of exposure

In the systematic evaluation of green space users’ likelihood of tick bites and subsequently acquiring TBPs, population density around the green space, visitor count, the proportion of green space users engaging in high-risk activities, and scores of knowledge, attitude, and practice concerning tick-borne diseases will be considered for the assessment of exposure.

### Population density data

The population census data for Bonn and Cologne will be sourced from the respective city administrations. Using this dataset, the population density will be estimated for 1km^2^ (to approximate potential daily visitors) and 9km^2^ (to approximate potential weekend visitors) around the centroid of the sampling spots in each green space. This methodology follows the approach reported by Janzén et al. (2024) [25].

### Visitor count (V)

Since there is no official recording of the number of visitors to the specific green spaces, the number of people and dogs visiting each green space will be counted during each tick collection. Visitor counts will be counted in a 20-minute period at the most trafficked areas of the green space, such as parking spots, playgrounds, or wildlife enclosures every month from March to October after the tick surveillance. The coordinates and characteristics of the most trafficked area, where visitors will be counted, are detailed in Additional File 1. To provide a uniform measure of human presence across UGS, the visitor count (V) recorded over the 20-minute observation period will be extrapolated to estimate the total number of visitors visiting a green space daily.

### In-field survey

Assessing the human-tick interactions is a crucial step for a comprehensive understanding of the risk of TBP transmission in UGS. The risk of exposure to ticks and potential transmission of TBPs at a UGS is influenced by the green space users: – the type of activities performed, green space users’ knowledge of TBDs, perceived risk of tick bites, adoption of preventive behaviours, and history of tick-bites at the green space. To understand the susceptibility of green space users to tick bites, an in-field survey will be conducted with green space users to assess their usage patterns, knowledge, risk perception, and preventative behaviours specific to the green space they were using at the time of the survey. The selection and enrolment of participants, along with the calculation of the required participant count, are detailed in Table 10. The variables assessed for each category in the in-field survey are described in Table 11. The questionnaire for the in-field survey can be found in the additional information section (see Additional File 2).

**Table 10:** Eligibility Criteria, participant recruitment and sample size for the In-field survey

Eligibility Criteria: Participants must be 18 or older to be included in the study. No additional exclusion criteria will be applied during the selection of respondents.
**Participant Recruitment**: Individuals will be approached in green spaces and invited to participate in the research study. Participants will be free to withdraw from an interview at any time without incurring any damages. The participation was anonymous in nature.
**Sample Size calculation**: The sample size for the Infield survey is calculated using the formula, Required sample size = [z^2^ * p(1-p)] / e^2^ a. z = Z-score. For the research study, it is 1.96, corresponding to the 9% confidence interval value. b. e = margin of error. For the research study, it is ± 5% c. p. = standard of deviation. For the research study, it is 0.5 For the total population of 1.4 million inhabitants for the cities of Cologne and Bonn, the study population was calculated to be ≈ 385 people. Among the 11 green spaces, it is expected to obtain 105, 140, and 140 surveys, respectively, in 3 categories: (i) Forest (*N* = 3), (ii) green space in the vicinity of/connected to a forest (*N* = 4), and (iii) green space far away from/separated from the forest (*N* = 4) of green spaces.

**Table 11:** Variables assessed in the in-field survey

Green space usage^1^	Knowledge	Attitude	Preventative behaviour^2^	History of tick-bite	Socio-demographic data
- The frequency of green space usage - Average time spent - Activities performed	- Awareness of preventative measures they adopt at the green space - Response to a tick-bite - Early signs of Lyme disease	- The severity of tick-borne diseases in Germany and the Bonn-Cologne region - Probability of getting bitten by a tick at the green space - Probability of getting infected with TBD after a tick-bite at the green space - Locations in the green space that contain ticks - Change in usage patterns due to ticks	- Long Clothes (During)* - Light Clothes (During)* - Pants tucked into socks (During)* - Usage of Insecticide or tick repellants (Before) - Frequency of visiting areas with higher grass/bushes or wilderness (During) - Probability of checking for ticks (After)	- History of tick-bite at the green space in the last 12 months and any reported tick-borne illness - History of Dog’s exposure to tick bite at the green space in the last 12 months and any reported tick-borne illness	- Gender - Age - Children below 13 years in the household - Ownership of one or more dogs - Highest education achieved - Net household income

1-The date of the Interview and the Interview location (the green space used at the time of the interview) were noted at the start of the interview. 2- Before/During/After the usage of green space on the day of the Interview. *- Observed by the interviewer during the interview

### Statistical analysis

Descriptive statistics will provide insight into the variables assessed in the in-field survey data: frequencies and percentages for categorical variables, mean values ± standard deviation for normally distributed continuous variables, and median with interquartile range for non-normal data. Group differences will be assessed with a chi-square test or Fisher’s exact probability test for categorical variables and T-tests or ANOVA for continuous outcome variables. Knowledge, Attitude, and Practice scores will be computed as respective indices. The relationships between knowledge, risk perception, and practice scores will be examined using correlation analysis (Pearson or Spearman), followed by regression analysis of knowledge and attitude scores against practice scores if a significant correlation exists. Correlation analysis will be conducted using Pearson or Spearman coefficients to examine associations between socio-demographic factors (age, gender, level of education, net household income, status of children below the age of 13 in the household, nationality), history of tick bites, green space usage, and type of green space with knowledge, risk perception, and preventative behaviours. Univariable regression analysis will be performed, and variables with P-values ≤ 0.25 will be selected for multiple regression analyses [79]. The results will be reported using regression coefficients, 95% confidence intervals (CI), and p-values. Statistical significance will be set up at *p* < 0.05.

## Integration of hazard and exposure to characterise risk at UGS

The risk of acquiring TBPs in UGS will be estimated by integrating entomological hazard and exposure. The risk can be estimated, as Janzén et al. (2024) reported, by summing the ordinal values (1 (low), 2 (Moderate) and 3 (High)) of the tick hazard (abundance of ticks, prevalence of TBPs) and the human exposure (visitor numbers) [25]. Alternatively, human health risks of green spaces can be calculated in terms of “weighted infection risk”, as suggested by Sormunen et al. (2020), factoring in the hazard, visitor count, and population density [21]. Weighted infection risk was calculated as Hazard × estimated visitor counts at the green space or Hazard × estimates of population density. However, the current research study intends to integrate the activity and preventive behaviour of green space users in the UGS into the assessment of exposure.

The risk (R) of acquiring a tick-borne infection in a green space will be estimated as a function of hazard (H) and exposure (E) using the formula, R = H × E, as modelled in risk assessment studies [80, 81]. Hazard (H) will be calculated as the density of infected tick specimens, calculated by multiplying the density of ticks per 100m^2^ (DOT) and the infection prevalence (IP), i.e. H_i_= DOT_i_ x IP_i_, where i is respective green space, DOT is the tick density per 100m^2^ and IP is the infection prevalence [6, 21]. Exposure (E) to ticks will be modelled as a function of visitor counts (V) and variables assessed in the In-field survey, the type of activity performed (Activity Score, A), the practice of preventative behaviour (PB), and self-reported incidents of tick bites for humans and dogs visiting the UGS (SRTB). The Activity Score (A) will be calculated by summing the weighted contributions of different activities performed by visitors (with high-risk activities assigned higher weight), considering the proportion of participants doing each activity, their visit frequency, and the average time spent per visit. The Preventative Behaviour Score (PB) will be calculated as the average proportion of visitors engaging in different preventative behaviours, with all behaviours weighed equally. Exposure will be estimated with the formula, E_i_ = V_i_ × A_i_ × (1 − PB_i_), where i is the respective green space. The self-reported tick bite incidents (SRTB) will be used to validate and adjust the formula.

Hazard and exposure, determined as H_i_= DOT_i_ x IP_i_ and E_i_ = V_i_ × A_i_ × (1 − PB_i_) respectively, will be integrated to estimate the risk score (R_i_) for each green space,

$$\eqalign{\>{{\rm{R}}_{\rm{i}}}{\rm{ = }}\> & ({\rm{DO}}{{\rm{T}}_{\rm{i}}}\> \times \>{\rm{I}}{{\rm{P}}_{\rm{i}}})\> \cr & \times ({{\rm{V}}_{\rm{i}}}\> \times \>{{\rm{A}}_{\rm{i}}} \times \>({\rm{1 - P}}{{\rm{B}}_{\rm{i}}})), \cr} $$

where i is the respective green space, DOT is the density of ticks per 100 m^2^, IP is the infection prevalence among ticks, V is the visitor count, A represents the activity score, and PB is the preventative behaviour score.

A multiple regression analysis will be conducted using the calculated risk score as the dependent variable to identify factors influencing the risk scores. Explanatory variables will include ecological and socio-behavioural factors such as green space type, micro-climatic variables, landscape connectivity and composition, host composition, knowledge scores, attitude scores, and history of tick-bites. The results will be reported as regression coefficients, 95% confidence intervals, and p-values, allowing for a robust assessment of the ecological and socio-behavioural determinants of TBP infection risk in UGS.

By integrating the hazard and exposure data, ecological and socio-behavioural risk maps for the region of Bonn-Cologne will be designed to visualise spatial data featuring the presence, abundance, and distribution of ticks and tick-borne pathogens and the risk of transmission of infections due to usage patterns, knowledge, risk perceptions, and preventative behaviours adopted at various green spaces through projected risk predictions [82].

## Discussion

Systemic collection, analysis, and dissemination of data related to infections of importance to public health is critical to disease surveillance and necessary for disease management [83]. The research will provide a holistic approach to assessing the risk of acquiring tick-borne infections in urban areas. It will address knowledge gaps in public health implications of TBPs in urban areas by providing a comprehensive understanding of tick abundance, the prevalence of TBPs, and its influencing factors, as well as factors affecting human exposure. To answer research questions 1 and 2, the study will assess the entomological hazard of tick bites in UGS, and understand the risk posed by ticks as disease vectors in UGS. Further, it will evaluate the critical elements of hazard assessment such as tick populations’ abundance, distribution, infection rates with pathogens, host associations, and environmental factors impacting these dynamics. Research question 3 will be addressed in the research study with the assessment of exposure of green space users to TBPs by conducting a KAP survey in the UGS. Exposure will be modelled as a function of visitor count, activities performed in the green space, and preventative behaviours practised by green space users. The risk factors associated with tick exposure in the green space will be characterised. Additionally, the research study will employ a quantitative risk assessment model to estimate the likelihood of human exposure to TBPs. The data obtained from entomological surveillance and exposure assessment will be integrated to calculate the risk of acquiring tick-borne infections in various UGS. This will provide a comprehensive framework for understanding the ecological and behavioural factors influencing tick-borne infection risks in urban environments. Last, the research study aims to provide city administrations with evidence-based public health strategies and recommendations for urban planning.

The study has certain limitations: The key limitation is the focus on characterising the risk of acquiring tick-borne infections instead of tick-borne diseases. The disease development after acquiring an infection depends on several factors, such as immunity, awareness of tick-borne diseases among the public and medical practitioners, and availability of effective medical diagnostics, which is not covered in this research study. Secondly, urban green spaces are complex socio-ecological systems, and numerous interacting factors influence the risk of acquiring tick-borne infections. As with any model, our risk score simplifies a complex and interacting system and cannot capture all factors influencing tick-borne infection risk in urban green spaces. To address this, we employ multiple regression analysis to investigate the impact of ecological and behavioural variables on risk. Still, in line with “all models are wrong, but some are useful,” the model should be seen as a comparative tool, not a precise predictor. Another limitation is the sole focus on human health risks, excluding animals and livestock from consideration, which are also affected by tick-borne diseases – leaving a gap in understanding the broader implication of tick-borne infections in the One Health context.

## Supplementary Information

Below is the link to the electronic supplementary material.

Supplementary Material 1: Coordinates of trafficked location in UGS where visitor counts will be done (PDF)

Supplementary Material 2: Questionnaire for In-field survey - German version (PDF)

Supplementary Material 3: Questionnaire for In-field survey - English version (PDF)

## Data Availability

The data collected as part of this research study will be available to the personnel of the GreenBalance project at the Institute of Hygiene and Public Health, University Hospital Bonn. Researchers who fulfil the criteria to access the data collected during the research study may contact Dr Timo Falkenberg, Principal Investigator, GreenBalance Projekt, Institut für Hygiene und öffentliche Gesundheit University Hospital Bonn, Gebäude 63 Venusberg Campus 1 53127 Bonn Tel. 0228-287- 19517, [timo.falkenberg[at]ukbonn.de](mailto: timo.falkenberg@ukbonn.de) .

## Abbreviations

A: Activity Score
B: Borrelia
DIT: Density of infected ticks/100m²
DNA: Deoxyribonucleic acid
DOT: Density of ticks/100m²
E: Exposure
H: Hazard
I: Ixodes
IP: Infection prevalence
KAP: Knowledge, Attitude, Practice
LB: Lyme Borreliosis
min: Minutes
NRW: North Rhine-Westphalia
PB: Preventative behaviour
PCR: Polymerase chain reaction
qPCR: Real-time Polymerase chain reaction
R: Risk
RNA: Ribonucleic acid
SRTB: Self-reported tick bites
TBD(s): Tick-borne disease(s)
TBE: Tick-borne encephalitis
TBP(s): Tick-borne pathogen(s)
sec: Second
UGS: Urban Green Space
V: Visitor Count
