# Supplementary material for: Assessment of human health risks to tick-borne infections in urban green spaces (UGS) - a study protocol
Source: BMC Infect Dis. 2025 Dec 24;26:170. doi: 10.1186/s12879-025-12364-6 (PMC12849638; doi:10.1186/s12879-025-12364-6)
Supplement: Supplementary file 1 — Supplementary Material 1: Coordinates of trafficked location in UGS where visitor counts will be done (PDF) [file 12879_2025_12364_MOESM1_ESM.pdf]

**Green Space**	**Visitor Counting spot**	**Characteristics**
**Bonn**		
Siebengebirge	50°39'46.4"N 7°13'10.5"E	Parking Area
Ennert	50°44'18.0"N 7°09'13.1"E	Entrance of the Green Space
Poppelsdorf Schloss/Area	50°43'34.9"N 7°05'37.2"E	In proximity to a water body visited by people
Kottenforst	50°40'11.4"N 7°04'29.5"E	Parking Area
Waldau	50°41'26.6"N 7°05'42.8"E	Animal enclosure
Rheinaue park	50°42'44.4"N 7°08'22.5"E	Nearest Tram Station leading to the Park
**Köln**		
Stadtwald	50°55'39.5"N 6°52'45.2"E	In proximity to a water body visited by people
Lindenthaler Tierpark	50°55'43.9"N 6°53'44.0"E	Animal enclosure
Aachener Weiher	50°56'08.0"N 6°55'44.5"E	In proximity to a water body
Gut Leidenhausen	50°53'34.6"N 7°05'33.9"E	Playing spot for kids
