# Supplementary material for: Assessment of human health risks to tick-borne infections in urban green spaces (UGS) - a study protocol
Source: BMC Infect Dis. 2025 Dec 24;26:170. doi: 10.1186/s12879-025-12364-6 (PMC12849638; doi:10.1186/s12879-025-12364-6)
Supplement: Supplementary file 2 — Supplementary Material 2: Questionnaire for In-field survey - German version (PDF) [file 12879_2025_12364_MOESM2_ESM.pdf]

Vor-Ort Befragung 18.04.2024

Einverständniserklärung

Einverständniserklärung für die Umfrage

"Vor-Ort Erhebung: Auswirkungen der Landschaftszusammensetzung und -vernetzung auf die Zeckenprävalenz, die Pathogendynamik und menschliche Gesundheitsrisiken in städtischen Grünflächen".

Mit Ihrer mündlichen Zustimmung zur Teilnahme an der Umfrage erklären Sie:

o Sie sind 18 Jahre alt oder älter.

o Die Art, die Bedeutung und der Umfang der Forschungsstudie wurden Ihnen vom Interviewer mündlich mitgeteilt. Außerdem wurden Sie über die möglichen Risiken und Vorteile einer Teilnahme an der Studie aufgeklärt.

o Sie sind über die Nutzung, Verarbeitung und den Schutz Ihrer Daten informiert. Sie nehmen zur Kenntnis, dass die Daten auf elektronischen Datenplattformen auf dem Server des Uniklinikums Bonn erhoben und verarbeitet werden können, ohne dass eine Weitergabe an Dritte erfolgt.

o Sie verstehen, dass Sie Ihre Einwilligung der Nutzung der Daten jederzeit widerrufen können. Im Falle des Widerrufs werden keine weiteren Daten erhoben.

o Sie erklären sich mit der Teilnahme an der Umfrage einverstanden.

Vom Interviewer beobachtete Informationen

A.Date of Interview

yyyy-mm-dd

B. In welcher Grünflache wurde die Umfrage durchgeführt?

- ◯ Kottenforst
- ◯ Venusberg
- ◯ Baumschulwäldchen-Hofgarten
- ◯ Siebengebirge (Rhöndorf)
- ◯ Ennert
- ◯ Beueler Rheinaue Park
- ◯ Rheinaue Park
- ◯ Stadtwald
- ◯ Lindenthaler Tierpark
- ◯ Uniwiese südlich des Aachener Weiher
- ◯ Gut Leidenhausen

C. Der/Die Befragte trägt lange Kleidung.

- ◯ Ja
- ◯ Nein

D. Der/Die Befragte trägt helle Kleidung.

- ◯ Ja
- ◯ Nein

E. Der/Die Befragte hat seine Hose in die Socken gesteckt.

- ◯ Ja
- ◯ Nein

Der folgende Abschnitt enthält Fragen zu Ihrer Nutzung dieser Grünfläche.

1. Wie oft besuchen Sie die Grünfläche, in der wir uns derzeit befinden?

- ◯ Mehr als 3 Mal pro Woche
- ◯ 1-3 Mal pro Woche
- ◯ 1-3 Mal pro Monat
- ◯ 1-3 Mal pro Jahr
- ◯ Erstes Mal

2. Wie lange nutzen Sie diese Grünfläche im Durchschnitt pro Besuch? (Bitte schreiben Sie die Antwort in Minuten.) (Offene Frage)

____

3. Welchen Aktivitäten gehen Sie auf dieser Grünfläche nach? (Mehrfachantworten möglich)

- ☐ Joggen
- ☐ Radfahren/Skaten
- ☐ Fitness/Gewichtstraining
- ☐ Mannschaftssportarten
- ☐ Gymnastik/Kraftsport
- ☐ Tiere beobachten/Landschaft betrachten
- ☐ Sonnenbaden
- ☐ Kinder unterhalten
- ☐ Mit Haustieren spazieren/spielen
- ☐ Spazierengehen
- ☐ Wandern
- ☐ Sitzen
- ☐ An Wasserfläche verweilen
- ☐ Freunde/Familie treffen
- ☐ Lesen/Schreiben
- ☐ Meditation
- ☐ Musik hören/machen
- ☐ Ausruhen/Schlafen
- ☐ Picknick/Grillen
- ☐ Sammlung von Kräuter/Pflanzen/Pilze/Holz
- ☐ Andere Tätigkeiten

Welchen andere Aktivitäten gehen Sie auf dieser Grünfläche nach?

____

Der folgende Abschnitt enthält Fragen zu Zecken und zeckenübertragenen Krankheiten in Grünanlagen.

4. Sind Sie der Meinung, dass zeckenübertragene Krankheiten in Deutschland ein ernstes Problem darstellen?

- ○ Sehr ernst
- ○ Ernst
- ○ Neutral
- ○ Geringfügig ernst
- ○ Gar nicht ernst

5. Sind Sie der Meinung, dass zeckenübertragene Krankheiten in der Region Köln-Bonn ein ernstes Problem darstellen?

- ◯ Sehr ernst
- ◯ Ernst
- ◯ Neutral
- ◯ Geringfügig ernst
- ◯ Gar nicht ernst

6. Für wie wahrscheinlich halten Sie es, dass Sie in dieser Grünfläche von einer Zecke gebissen werden?

- ◯ Sehr wahrscheinlich
- ◯ Ziemlich wahrscheinlich
- ◯ Neutral
- ◯ Eher unwahrscheinlich
- ◯ Sehr unwahrscheinlich

7. Falls Sie auf dieser Grünfläche von einer Zecke gebissen werden: Für wie wahrschienlich halten Sie es, sich mit einer zeckenübertragenen Krankheit anzustecken?

- ◯ Sehr wahrscheinlich
- ◯ Ziemlich wahrscheinlich
- ◯ Neutral
- ◯ Eher unwahrscheinlich
- ◯ Sehr unwahrscheinlich

8. An welchen Orten in dieser Grünfläche erwarten Sie, von Zecken gebissen werden zu können? (Mehrfachantworten möglich)

- ☐ Gemähter Rasen
- ☐ Ungemähter Rasen
- ☐ An kleinen Büschen am Rande der Wege
- ☐ Im trockenen Laub und Todholz
- ☐ unter Bäumen
- ☐ Keine
- ☐ Andere

Welche anderen Stellen in dieser Grünfläche könnten Ihrer Meinung nach Zecken enthalten?

____

» 9. Geben Sie für jede der folgenden Aussagen zu dieser Grünfläche an, ob sie richtig oder falsch sind.

	Ja	Nein
**a) Sie haben die Aktivitäten, die Sie auf dieser Grünfläche ausüben wegen des Zeckenvorkommens geändert.**	◯	◯

b) Sie nutzen diese Grünfläche, um andere Grünflächen mit höherem Zeckenvorkommen zu vermeiden. ○ ○

c) Aufgrund der Gefahr eines Zeckenbisses benutzen Sie diese Grünfläche nicht so oft. ○ ○

9. Welche Vorkehrungen zur Zeckenabwehr treffen Sie vor/ während/nach der Nutzung dieser Grünfläche? (Mehrfachantworten möglich) (Offene Frage)

- ☐ Insektenschutzmittel auf Haut oder Kleidung auftragen
- ☐ Helle, lange Kleidung tragen
- ☐ Auf den Wegen bleiben, um hohe Gräser und Sträucher zu vermeiden
- ☐ Hosen in die Socken stecken
- ☐ Innerhalb von zwei Stunden nach der Rückkehr auf Zecken absuchen und/oder duschen
- ☐ Keine
- ☐ Andere

Welche andere Vorkehrungen treffen Sie vor/während/nach der Nutzung dieser Grünfläche? (Offene Frage)

____

10. Haben Sie heute vor dem Besuch dieser Grünfläche Insekten- oder Zeckenschutzmittel auf Ihre Haut oder Kleidung aufgetragen?

- ○ Ja
- ○ Nein

11. Wie oft gehen Sie in die Bereiche mit hohem Gras und/oder Sträuchern und/oder in den Waldbereich, wenn Sie diese Grünfläche nutzen?

- ○ Sehr oft
- ○ Häufig
- ○ Manchmal
- ○ Selten
- ○ Niemals

12. Wie wahrschheinlich ist es, dass Sie sich nach einem Besuch dieser Grünflache zu Hause nach Zecken absuchen?

- ○ Sehr wahrscheinlich
- ○ Ziemlich wahrscheinlich
- ○ Neutral
- ○ Eher unwahrscheinlich
- ○ Sehr unwahrscheinlich

13. Was würden Sie tun, wenn Sie von einer Zecke gebissen werden? (Mehrfachantworten möglich) (Offene Frage)

- ☐ Ich trage Chemikalien (Vaseline, Cream usw...) auf und töte die Zecke damit ab, bevor Ich sie entferne.
- ☐ Ich zupfe die Zecke mit den Fingern aus der Haut.
- ☐ Ich verwende eine Pinzette, um die Zecke zu entfernen.
- ☐ Ich verwende eine Zeckenkarte, um die Zecke zu entfernen.
- ☐ Ich verwende ein Desinfektionsmittel, nachdem ich die Zecke entfernt habe.
- ☐ Ich suche ärztlichen Rat.
- ☐ Ich nehme Antibiotika ein.
- ☐ Ich weiß es nicht.
- ☐ Ich handele ganz anders.

Was würden Sie sonst tun, wenn Sie von einer Zecke gebissen würden?

____

14. Ein Frühanzeichen der Lyme-Borreliose ist : (Mehrfachantworten möglich) (Offene Frage)

- ☐ Ausschlag
- ☐ Fieber
- ☐ Ermüdung
- ☐ Müdigkeit
- ☐ Muskelschmerzen
- ☐ Gelenkschmerzen
- ☐ Erythema migrans (Wanderröte)
- ☐ Ich weiß es nicht.
- ☐ Andere

Ein Frühanzeichen der Lyme-Borreliose ist : (Oben nicht erwähnt)

____

15. Wurde Sie in den letzten 12 Monaten in dieser Grünfläche von einer Zecke gebissen?

- ○ Ja
- ○ Nein

Wie oft wurden Sie auf dieser Grünfläche von einer Zecke gebissen?

____

Sind Sie durch den Zeckenbiss erkrankt?

- ○ Ja
- ○ Nein
- ○ Ich weiß es nicht

Welche Krankheit

Wie lange hat es gedauert, bis ein Arzt die Diagnose gestellt hat?
Geben Sie bitte die Anzahl der Tage an.

---

16. Haben Sie einen oder mehrere Hunde?

- ◯ Ja
- ◯ Nein

Wie viele?

Wurde in den letzten 12 Monaten einer Ihrer Hunde von einer Zecke gebissen?

- ◯ Ja
- ◯ Nein
- ◯ Ich weiß es nicht

Wie oft wurde der betroffener Hund von einer Zecke gebissen? Sofern mehr als ein Hund gebissen worden ist, geben Sie bitte die durchschnittliche Anzahl der Zeckenbisse pro Tier an.

Hat sich einer Ihrer Hunde nach dem Zeckenbiss mit einer Krankheit infiziert?

- ◯ Ja
- ◯ Nein
- ◯ Ich weiß es nicht

Welche Krankheit?

Wie lange hat es gedauert, bis ein Arzt die Diagnose gestellt hat?
Geben Sie bitte die Anzahl der Tage an.

Nutzen Sie einen Zeckenschutz für Ihren Hund?

- ◯ Ja
- ◯ Nein

Welche?

Demografische Angaben

17. Mit welchem Geschlecht identifizieren Sie sich?

- ◯ Weiblich
- ◯ Männlich
- ◯ Divers
- ◯ Keine Angabe erwünscht

18. Wie alt sind Sie?

- ◯ 18-29
- ◯ 30-39
- ◯ 40-49
- ◯ 50-59
- ◯ 60-69
- ◯ 70-79
- ◯ 80 oder älter
- ◯ Keine Angabe erwünscht

19. Leben Ihre Kinder oder Enkelkinder unter 13 Jahren in Ihrem Haushalt? (Mehrfachantworten möglich)

- ☐ Ja (Kinder)
- ☐ Ja (Enkelkinder)
- ☐ Nein
- ☐ Keine Angabe erwünscht

Wie viele Kinder haben Sie?

____

Wie viele Enkelkinder haben Sie?

____

20. Was ist Ihr höchster Bildungsabschluss?

- ◯ Kein Schulabschluss
- ◯ Hauptschulabschluss
- ◯ Realschule (Mittlere Reife)
- ◯ Gymnasium (Abitur)
- ◯ Abgeschlossene Ausbildung
- ◯ Fachhochschulabschluss
- ◯ Hochschulabschluss
- ◯ Anderer Bildungsabschluss
- ◯ Keine Angabe erwünscht

Welchen anderen Bildungsabschluss haben Sie?

____

21. Wie hoch ist Ihr monatliches Nettohaushaltseinkommen?

- ◯ Unter 500€
- ◯ 500€ bis unter 1.000€
- ◯ 1.000€ bis unter 2.000€
- ◯ 2.000€ bis unter 3.000€
- ◯ 3.000€ bis unter 4.000€
- ◯ 4.000€ bis unter 5.000€
- ◯ 5.000€ oder mehr
- ◯ Keine Angabe erwünscht

Herzlichen Dank! Sie sind am Ende des Fragebogens angekommen. Vielen Dank für Ihre Teilnahme!
