# Supplementary material for: Assessment of human health risks to tick-borne infections in urban green spaces (UGS) - a study protocol
Source: BMC Infect Dis. 2025 Dec 24;26:170. doi: 10.1186/s12879-025-12364-6 (PMC12849638; doi:10.1186/s12879-025-12364-6)
Supplement: Supplementary file 3 — Supplementary Material 3: Questionnaire for In-field survey - English version (PDF) [file 12879_2025_12364_MOESM3_ESM.pdf]

On-site survey 18.04.2024

Declaration of consent

Declaration of consent for the survey

‘On-site survey: Effects of landscape composition and connectivity on tick prevalence, pathogen dynamics and human health pathogen dynamics and human health risks in urban green spaces’.

By verbally agreeing to participate in the survey, you declare that

- You are 18 years of age or older.
- You have been informed verbally by the interviewer of the nature, significance and scope of the research study. You have also been informed about the possible risks and benefits of participating in the study.
- You have been informed about the use, processing, and protection of your data. You acknowledge that your data can be collected and processed on electronic data platforms on the University Hospital Bonn server without being passed on to third parties.
- You understand that you can revoke your consent to the use of the data at any time. In the event of revocation, no further data will be collected.

You agree to participate in the survey.

- o Yes
- o No

The interviewer proceeds with the survey after the interviewee agrees to participate.

Information observed by the interviewer

A: Date of the Interview

yyyy-mm-dd

B. In which green space was the survey conducted?

- o Kottenforst
- o Venusberg
- o Baumschulwäldchen-Hofgarten
- o Siebengebirge (Rhöndorf)
- o Ennert
- o Beueler Rheinaue Park
- o Rheinaue Park
- o Stadtwald
- o Lindenthaler Tierpark
- o Uniwiese südlich des Aachener Weiher
- o Gut Leidenhausen

C. The interviewee wears long clothing.

- o Yes
- o No

D. The interviewee wears light-coloured clothing.

- o Yes
- o No

E. The interviewee has tucked their trousers into their socks.

- o Yes
- o No

The following section contains questions about your use of this green space.

1. How often do you visit the green space we are currently in?

   - o More than 3 times a week
   - o 1-3 times a week
   - o 1-3 times per month
   - o 1-3 times per year
   - o First time

2. On average, how long do you use this green space per visit? (Open question)

   ____minutes.

3. What activities do you do in this green space? (multiple answers possible)

   - o Jogging
   - o Cycling/Skating
   - o Fitness/weight training
   - o Team sports
   - o Gymnastics/weight training
   - o Observing animals/viewing the landscape
   - o Sunbathing
   - o To entertain children
   - o Walking/playing with pets
   - o Going for a walk
   - o Hiking
   - o Sitting
   - o Visiting Waterbodies
   - o Meeting friends/family
   - o Reading/writing
   - o Meditation
   - o Listening to/making music
   - o Resting/sleeping
   - o Picnic/barbecue
   - o Collection of herbs/plants/mushrooms/wood

- Other activities

If “Other activities”, what other activities do you do in this green space? (Open Question)

The following section contains questions on ticks and tick-borne diseases in green spaces.

4. Do you think that tick-borne diseases are a serious problem in Germany?

   - Very serious
   - Serious
   - Neutral
   - Slightly serious
   - Not serious at all

5. Do you think that tick-borne diseases are a serious problem in the Cologne-Bonn region?

   - Very serious
   - Serious
   - Neutral
   - Slightly serious
   - Not serious at all

6. How likely do you think it is that you will be bitten by a tick at this green space?

   - Very likely
   - Quite likely
   - Neutral
   - Rather unlikely
   - Very unlikely

7. If you were bitten by a tick in this green space, how likely do you think it is that you will acquire a tick-borne disease?

   - Very likely
   - Quite likely
   - Neutral
   - Rather unlikely
   - Very unlikely

8. In which places in this green space do you expect to be bitten by ticks? (multiple answers possible)

   - Mowed lawn
   - Unmowed lawn
   - On small bushes at the edge of the paths
   - In dry leaves and deadwood under trees

- None
- Other

If “Other”, what other places in this green space do you think might contain ticks? (Open Question)

---

9. "For each of the following statements about this green space, indicate whether they are true or false.

a) You have changed the activities you carry out in this green space because of the risk of ticks.
b) You use this green space to avoid other green spaces with higher tick incidence.
c) Due to the risk of tick bites, you do not use this green space so often.

10. What precautions do you take to prevent ticks before/during/after using this green space? (multiple answers possible) (Open question)

- Apply insect repellent to skin or clothing
- Wear light colored, long clothing
- Stay on the paths to avoid high grass and bushes
- Tuck your pants into your socks
- Check for ticks and/or shower within two hours of returning from this green space.
- None
- Other

If “Other”, what other precautions do you take before/during/after using this green space? (Open question)

---

11. Did you apply insect or tick repellent to your skin or clothing before visiting this green space today?

- Yes
- No

12. How often do you go to the areas with tall grass and/or shrubs and/or to the wooded area when you use this green space?

- Very often
- Often
- Sometimes
- Rarely
- Never

13. How likely is it that you will check yourself for ticks at home after visiting this green space?

- Very likely

- Quite likely
- Neutral
- Rather unlikely
- Very unlikely

14. What would you do if you were bitten by a tick? (multiple answers possible) (open question)

- I apply chemicals (Vaseline, cream, etc.) and kill the tick before removing it.
- I pluck the tick out of the skin with my fingers.
- I use tweezers to remove the tick.
- I use a tick card to remove the tick.
- I use a disinfectant after I remove the tick.
- I seek medical advice.
- I use antibiotics.
- I don't know.
- Other

If “Other”, what else would you do if you were bitten by a tick?

---

15. An early sign of Lyme borreliosis is : (multiple answers possible) (open question)

- Rash
- Fever
- Fatigue
- Fatigue
- Muscle pain
- Joint pain
- Erythema migrans (migratory rash)
- I don't know.
- Other

An early sign of Lyme borreliosis is: (not mentioned above)

---

16. Have you been bitten by a tick in this green space in the last 12 months?

- Yes
- No

If “Yes”, how often have you been bitten by a tick in this green space?

If yes, were you infected with a tick-borne disease after the tick bite?

- Yes
- No
- I do not know

If “Yes”, which disease ______________________

If “Yes”, how long did it take for a doctor to make the diagnosis? Please state the number of days. ___________________________________

17. Do you have one or more dogs?

- Yes
- No

If “Yes”, how many? _________________

If “Yes”, have any of your dogs been bitten by a tick in the last 12 months?

- Yes
- No
- I do not know

If “Yes”, how often was the dog bitten by a tick? If more than one dog has been bitten, please state the average number of tick bites per animal. (Open Question)

________________________times.

If “Yes”, has one of your dogs contracted a disease after being bitten by a tick?

- Yes
- No
- I do not know

If “Yes”, What illness? _________________________

If “Yes”, how long did it take for a doctor to make the diagnosis? Please state the number of days.

____

If “Yes”, do you use tick protection for your dog?

- Yes

- No

If “Yes”, which ones? (Open Question) _________________________

Demographic data

17. Which gender do you identify with?

- Female
- Male
- Diverse
- Prefer not to say

18. How old are you?

- 18-29
- 30-39
- 40-49
- 50-59
- 60-69
- 70-79
- 80 or older
- Prefer not to say

19. Do you have any children or grandchildren under the age of 13 living in your household? (multiple answers possible)

- Yes (children)
- Yes (grandchildren)
- No
- Prefer not to say

If “Yes (children)”, how many children do you have? ________________

If “Yes (grandchildren)”, how many grandchildren do you have? __________________

18. What is your highest educational qualification?

- No school-leaving certificate
- Hauptschulabschluss (basic secondary schooling)
- Realschule (intermediate school leaving certificate)
- Gymnasium (Abitur)
- Completed vocational training
- University of Applied Sciences degree
- University degree
- Other educational qualification
- Prefer not to say

If “Other educational qualifications”, what other educational qualifications do you have?

____

19. What is your monthly net household income?

- Under 500€
- 500€ to under 1,000€
- 1,000€ to under 2,000€
- 2,000€ to under 3,000€
- 3,000€ to under 4,000€
- 4,000€ to under 5,000€
- 5,000€ or more
- Prefer not to say

Thank you very much! You have reached the end of the questionnaire. Thank you for your participation.
